# Initiation and promotion in the transitional epithelium of the rat bladder.

**DOI:** 10.1038/bjc.1980.84

**Published:** 1980-03

**Authors:** R. M. Hicks


### INITIATION AND PROMOTION IN THE TRANSITIONAL EPITHELIUM OF THE RAT BLADDER

R. M. HICKS

*From the School of Pathology, Middlesex Hospital Medical School, London*

THE THEORY of two-stage carcinogenesis involving initiation and promotion was developed by Berenblum (Berenblum, 1974) and the experimental data on carcinogenesis in mouse skin which he and his colleagues produced in the 1940s and 1950s still provide the factual basis which underpins current concepts of multi-stage carcinogenesis. Multistage models in which the promoting phase is further divided into two or more stages are required to explain the epidemiological data on time-related tumour incidence in man, and they also apply to experimental carcinogenesis both in skin and other organs, including the liver and bladder.

Multi-stage carcinogenesis in the bladder involves first a permanent transformation of cells in the transitional epithelium (urothelium) into latent tumour cells by a threshold or sub-threshold dose of a bladder carcinogen such as N-methyl-N-nitrosourea (MNU) N-butyl-N-(4-hydroxybutyl) nitrosamine (BBN) or N-(4-(5-nitro-2-furyl)-2-thiazolyl) formamide (FANFT). The initiated cells may then be converted into tumour cells by further exposure to the same or another carcinogen which is organotropic for the urothelium, or by prolonged application of a promoter which itself is not effective as a complete (initiating + promoting) carcinogen. In the bladder, saccharin, cyclamate, tryptophan and cyclophosphamide all behave predominantly as promoters. Development of a visible growing tumour from the promoted cells may then be accelerated by other hyperplastic agents such as bladder calculi which, though they may not be able to substitute for promoters in the early stages of conversion of an initiated cell into a latent tumour cell, will propagate subsequent tumour growth by stimulating the rate of cell turnover in the transformed urothelium.

Berenblum noted: 1. that for an unequivocal demonstration of a second, promoting stage of carcinogenesis, the initiating carcinogen must be used at a dose which is not carcinogenic to any marked degree; 2. that tumour incidence is related to the dose of initiator, not to the dose of promoter; 3. that initiation requires only brief exposure to the carcinogen and that the change produced is persistent; and 4. that promotion requires prolonged application of the promoter, and is reversible in its early stages.

These observations were made on the basis of mouse skin, but are also demonstrably true for carcinogenesis in the urinary bladder. Thus: 1. The effect of initiation with either MNU or FANFT, used at threshold or subthreshold doses, is promoted by subsequent prolonged feeding of saccharin or cyclamate in the diet (Hicks *et al.*, 1978; Cohen *et al.*, 1979). If, however, MNU is used at a dose which produces a 40% incidence of bladder tumours, no increase in tumour incidence is produced by ingestion of sweeteners, although in the same experiment a 28% incidence of urothelial tumours of the renal pelvis was promoted to 57% by saccharin and to 43% by cyclamate (Mohr *et al.*, 1978). This is directly analogous to observations made with mouse skin which showed that the tumour incidence following a high carcinogenic dose of benzo(a)pyrene could not be increased by subsequent application of the promoter croton oil (Berenblum, 1941). 2. In the bladder, after one particular sub-threshold initiating dose of MNU, the tumour incidence is constant at $\sim 50\%$ following promotion with either dietary saccharin or cyclamate, irrespective of the dose of sweetener (Hicks *et al.*, 1978). 3. For initiation with MNU, all that is required is a single, intravesicle installation of a low dose ($\sim 0{\cdot}2$ mg) which, because of the rate of spontaneous decomposition in the body, probably persists in the bladder for not more than 20–30 min. Initiation with MNU has now been demonstrated to persist for at least 6 months, and with FANFT for at least 6 weeks, though with the latter compound there is some reduction in the subsequent tumour incidence which follows promotion, suggesting the presence of effective excision repair in the urothelium. 4. No promotion was obtained with a single dose of cyclophosphamide after initiation with MNU (Hicks *et al.*, 1978) but prolonged dosing with cyclophosphamide promoted tumour growth following initiation with FANFT (Cohen *et al.*, 1979).

These findings, together with other experimental data now available, support the hypothesis that carcinogenesis in the urinary bladder, as in the mouse skin, is a multi-stage process involving initiation, promotion and propagation.

REFERENCES

BERENBLUM, I. (1941) *Cancer Res.*, **1**, 807.

BERENBLUM, I. (1974) In *Frontiers of Biology* 34. Ed. Neuberger & Tatum. Amsterdam and Oxford: North Holland.

COHEN, S. M., ARAI, M., JACOBS, J. B. & FRIEDELL, G. H. (1979) *Cancer Res.*, **39**, 1207.

HICKS, R. M., CHOWANIEC, J. & WAKEFIELD, J. ST. J. (1978) *Carcinogenesis, 2. Mechanisms of tumour promotion and co-carcinogenesis.* Ed. Slaga *et al.*, New York: Raven Press. p. 475.

MOHR, U., GREEN, U., ALTOFF, J. & SCHNEIDER, P. (1978) *Health and Sugar Substitutes* Proc. ERGOB Conference, Geneva. p. 64.


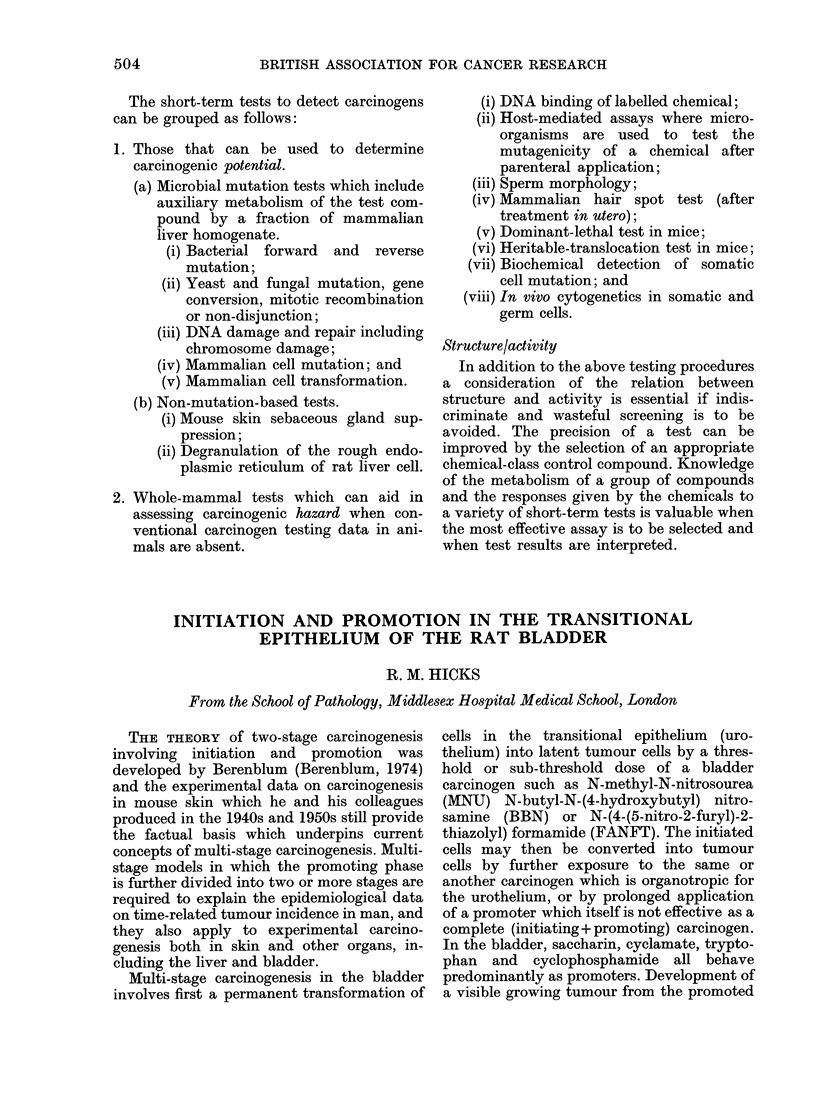

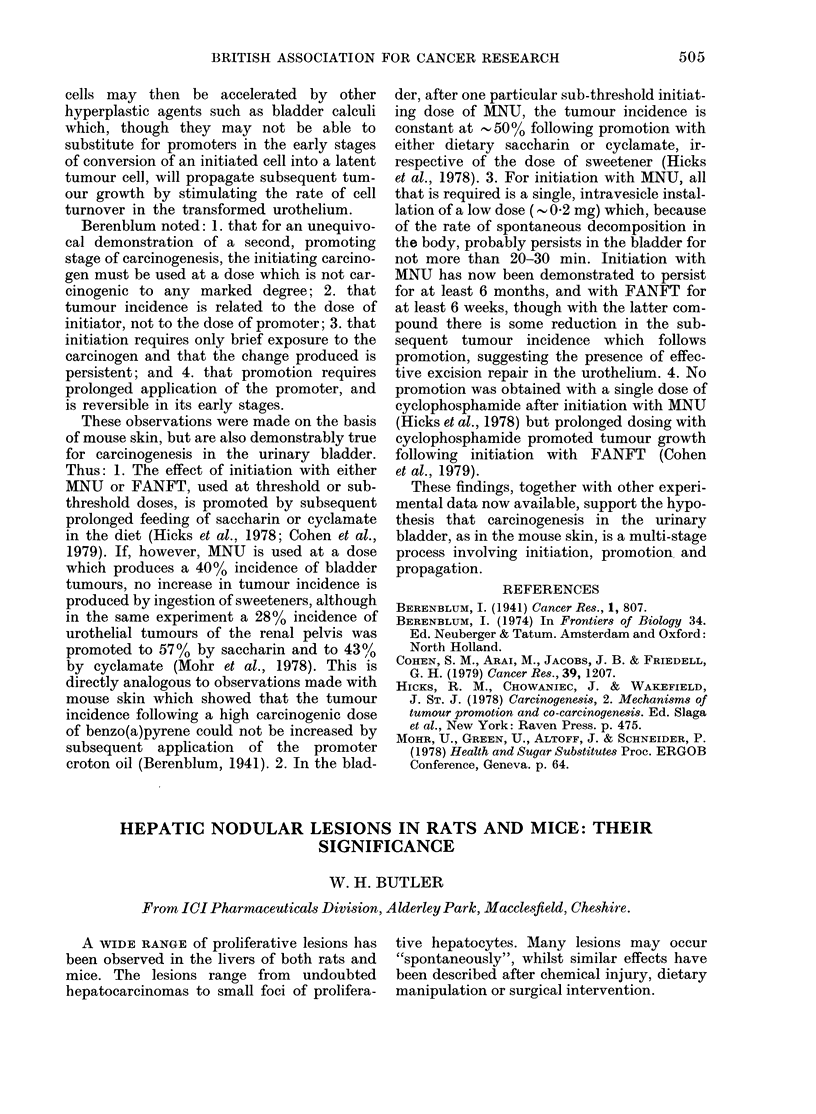


## References

[OCR_00135] Cohen S. M., Arai M., Jacobs J. B., Friedell G. H. (1979). Promoting effect of saccharin and DL-tryptophan in urinary bladder carcinogenesis.. Cancer Res.

